# Remote maintenance cardiac rehabilitation (MAINTAIN): A protocol for a randomised feasibility study

**DOI:** 10.1177/20552076231152176

**Published:** 2023-02-15

**Authors:** Francesca Denton, Alexander Waddell, Chris Kite, Katie Hesketh, Lou Atkinson, Matthew Cocks, Helen Jones, Harpal Randeva, Nathan Davenport, Richard Powell, Cain Clark, Ioannis Kyrou, Amy E Harwood, Gordon McGregor

**Affiliations:** 1 Institute of Health and Wellbeing, 2706 Coventry University, Coventry, UK; 2 Warwickshire Institute for the Study of Diabetes, Endocrinology and Metabolism (WISDEM), 2708 University Hospitals Coventry and Warwickshire NHS Trust, Coventry, UK; 3 School of Public Health Studies, Faculty of Education, Health and Wellbeing, University of Wolverhampton, Wolverhampton, UK; 4 School of Psychology, College of Health and Life Sciences, 1722 Aston University, Birmingham, UK; 5 Research Institute of Sport and Exercise Sciences, 4589 Liverpool John Moores University, Liverpool, UK; 6 Aston Medical School, College of Health and Life Sciences, 1722 Aston University, Birmingham, UK; 7 Laboratory of Dietetics and Quality of Life, School of Food and Nutritional Sciences, Department of Food Science & Human Nutrition, Agricultural University of Athens, Athens, Greece; 8 UK Department of Cardiopulmonary Rehabilitation, Centre for Exercise & Health, University Hospitals Coventry & Warwickshire NHS Trust, Coventry, UK; 9 Warwick Medical School, University of Warwick, Coventry, UK

**Keywords:** coronary heart disease, exercise maintenance, cardiac rehabilitation, wearable activity monitors, digital health, mHealth technology

## Abstract

**Background:**

Long-term adherence to exercise is often poor for people with coronary heart disease (CHD) who have completed supervised, centre-based cardiac rehabilitation. The aim of this study is to assess the feasibility of a remotely prescribed, delivered and monitored cardiac rehabilitation intervention using a wearable device to support long-term adherence to exercise and physical activity during maintenance of cardiac rehabilitation.

**Methods:**

After completing cardiac rehabilitation, 30 participants with CHD, will be randomised (1:1) to an intervention (*n* = 15) or a usual care group (*n* = 15) in a 12-month feasibility randomised controlled trial (RCT). The intervention will comprise of an exercise consultation, personalised exercise prescription delivered via a wearable activity monitor using biometric feedback, regular monitoring via check-ins, and feedback text-messages for 6-months. Participants will be assessed at baseline (following completion of cardiac rehabilitation) and at three-, six-, and 12-months post-randomisation. The primary outcome will be feasibility, including assessment of eligibility, recruitment, adherence, and acceptability. Secondary outcomes will include exercise capacity, physical activity behaviours, cardiovascular disease risk and quality of life. Semi-structured interviews will be conducted at three-, six-, and 12-months post-randomisation (and with those who drop-out) to explore the acceptability of the study intervention and procedures. A questionnaire will be offered to those who decline participation.

**Discussion:**

The MAINTAIN study will evaluate the feasibility of conducting a future definitive multi-centre RCT testing a remotely prescribed and monitored long-term mHealth maintenance exercise programme, versus usual care, for people with CHD who have completed cardiac rehabilitation.

**Trial registration number:**

ClinicalTrials.gov, NCT05292287. Registered on 22/03/2022

## Introduction

Approximately 2.3 million people in the UK are living with coronary heart disease (CHD), requiring long-term management and support.^1^ This costs in excess of £7 billion in the UK, highlighting not only a personal cost for those affected but also a substantial economic burden.^2^ Long-term management of CHD is multi-faceted, including a combination of medical, pharmacological and lifestyle interventions such as cardiac rehabilitation (CR),^3^ all of which are recommended by the National Institute for Health and Care Excellence (NICE).^4^

Home and centre-based exercise rehabilitation programmes can improve exercise capacity, cardiovascular disease risk markers, quality of life and psychological health in the short- to medium-term.^5,6^ Home-based CR is also considered a safe alternative to centre-based CR.^7-9^ However, physical activity (PA) and exercise discontinuation after supervised CR can have detrimental effects on health, leading to a higher risk of recurrent cardiovascular events, hospitalisation, and premature mortality.^10^ To prevent these negative health outcomes, discharge plans are created on completion of CR which include advice on maintaining PA and exercise as a key part of secondary prevention models.^11,12^ However, due to limited resources, long-term plans are often difficult to implement with direct supervision or follow-up. Consequently, long-term adoption of favourable PA and exercise behaviours is rarely successful, with sporadic maintenance of health-outcomes in cardiovascular disease.^13,14^

Whilst centre-based CR is “gold-standard” there are often barriers to attending, including time availability, transport, travelling distance, financial responsibilities, and work and family commitments.^15^ Alternatively, home-based CR may provide many benefits such as an increased capacity to deliver CR, the potential to personalise programs, and the ability to be more flexible with scheduling and to remove the barriers associated with travelling to CR centres.^7^ Furthermore, the COVID-19 pandemic and subsequent government-enforced lockdowns and social distancing measures have been detrimental to PA participation and almost half of CR services halted all rehabilitation.^16,17^ During this time, face-to-face CR was suspended, which increased the demand for remotely monitored and prescribed home-based exercise programs using telephone calls and/or live video conferencing, often referred to as mHealth.^17-19^ To improve the effectiveness of these programmes, wearable activity devices, providing biometric feedback on a range of variables, can be used to support personalised exercise prescription.^20,21^ These devices may also support improvements in cardiometabolic risk factors, such as inactivity, systolic blood pressure and low-density lipoprotein cholesterol.^22,23^ When combined with personalised text messages from health professionals, wearable activity monitors are often more effective at improving exercise and PA behaviour.^24,25^ Thus, remotely monitored and prescribed CR programs supported by wearable activity monitors may be a feasible alternative or adjunct to centre-based CR during pandemic scenarios.^21^ Two pilot randomised controlled trials (RCTs)^26,27^ reported the potential of wearable activity monitors to support PA maintenance, but only for a period of eight-12 weeks. Whilst wearable activity monitors have been associated with higher short term exercise adherence in clinical populations, longer-term data are sparse and less convincing.^28^ Thus, understanding the effectiveness of long-term home-based CR maintenance programmes using mHealth is ever more essential to overcome both traditional and recently emerging barriers to centre-based CR.

### Rationale for a randomised controlled trial

To improve quality of life and other health outcomes, emerging mHealth technologies may help to bridge the gap between CR teams and people with CHD in the long-term maintenance phase of CR. Assessing the ability of novel mHealth-assisted prescription and monitoring of personalised exercise may help to reduce secondary cardiovascular disease risk, when transitioning from supervised to independent exercise. Despite the potential for long-term benefit, it is equally important to consider the acceptability of these technologies from the perspective of the patient. Furthermore, previous research has not tested newer wearable devices which can ‘coach’ the user by remotely delivering exercise programmes in real-time, ensuring that the correct intensity, duration, and technique are achieved with the support of haptic feedback. This capacity to deliver immediate feedback to the user, whilst data are synchronised to an online web platform accessible to an exercise physiologist, may help people to maintain the PA and exercise behaviours achieved during CR.^29^ Consequently, for people with CHD, a feasibility RCT is needed to evaluate a long-term remotely prescribed and monitored exercise intervention supported by a wearable activity monitoring device providing real-time biometric feedback.

The reMote mAINTenance cArdiac rehabilItatioN (MAINTAIN) study aims to investigate the feasibility of conducting a full-scale RCT testing a remotely prescribed and monitored long-term maintenance exercise programme supported by mHealth, compared to usual care, for people who have recently completed supervised or home-based centre-based CR.

### Objectives

1. Conduct a randomised feasibility study with 30 participants randomised (1:1) to a remotely prescribed and monitored exercise intervention or usual care. Process-related measures will include eligibility, recruitment, and adherence to the intervention. Physiological, clinical, and health-related measures will include functional capacity and health-related quality of life (HRQoL).
2. Conduct a qualitative analysis to explore the views, perceptions, acceptability, and experiences of participants regarding the remotely prescribed and monitored exercise intervention and all trial procedures.

## Methods

### Study design

The MAINTAIN study is a single centre, assessor blind, feasibility RCT, including a qualitative sub-study. The Standard Protocol Items: Recommendations for Interventional Trials (SPIRIT) guidelines were followed to develop this protocol.^30^

### Study setting

**Figure 1.**
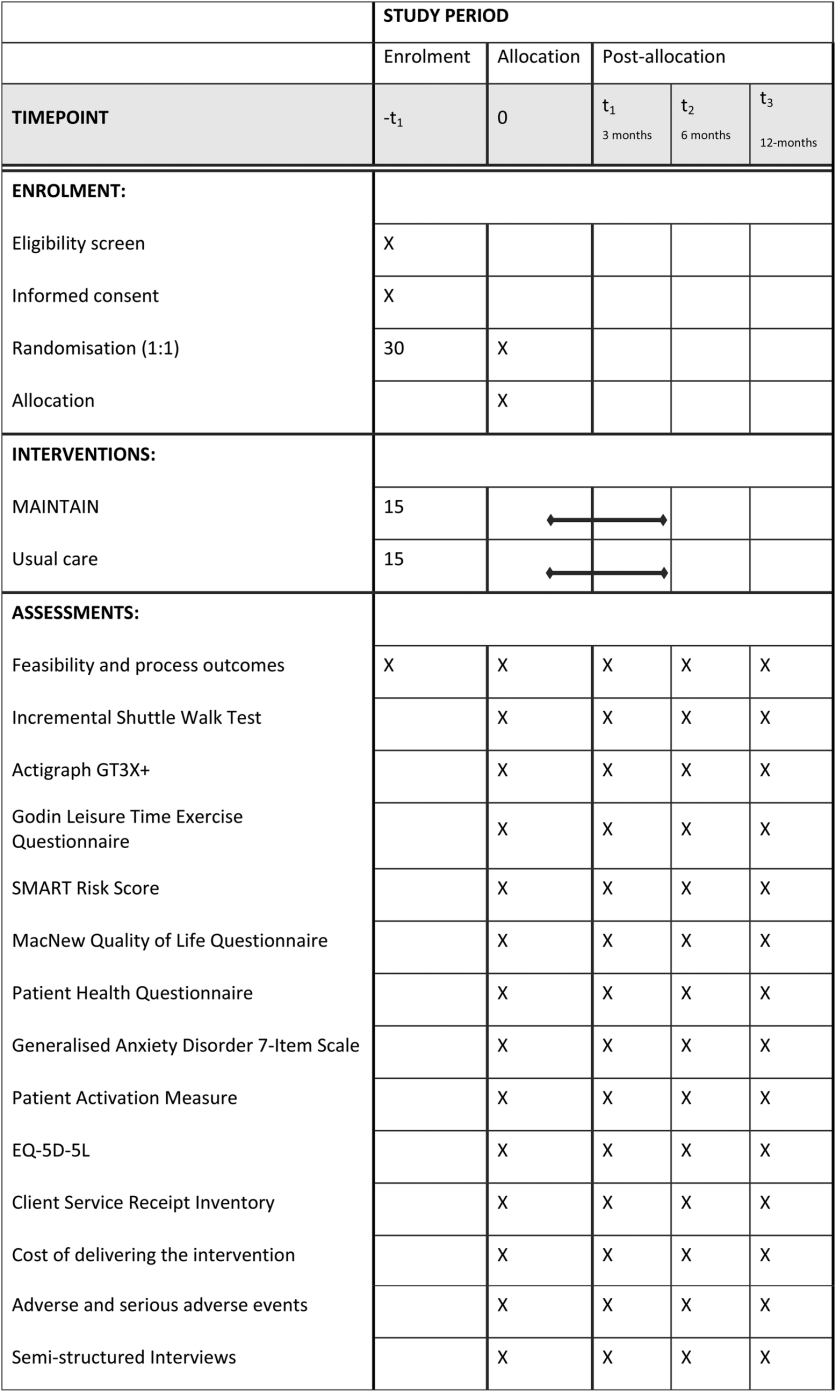
Schedule of enrolment, interventions, and assessments.

Recruitment will be carried out at an outpatient community CR centre at University Hospitals Coventry & Warwickshire (UHCW) NHS Trust (Figure 1). The study summary according to the World Health Organization (WHO) Trial Registration Data Set is outlined in Table 1.

**Table 1.** World health organization trial data set.

Data category	Information
Primary registry and trial identifying number	NCT05292287
Date of registration in primary registry	22/03/2022
Secondary identifying numbers	REC reference: 21/LO/0705 IRAS Number: 302243 Protocol number: P121990
Primary sponsor	Coventry University Priory Street Coventry CV1 5FB hls.rso@coventry.ac.uk
Secondary sponsor	N/A
Contact for public queries	Email: dentonf@uni.coventry.ac.uk
Contact for scientific queries	Francesca Denton Centre for Sport, Exercise and Life Sciences Research Institute for Health & Wellbeing Coventry University CV1 5FB Email: dentonf@uni.coventry.ac.uk
Public title	Long-term CR using remote exercise prescription and monitoring via a wearable device
Scientific title	reMote mAINTenance cArdiac rehabIlitatioN (MAINTAIN): a randomised controlled feasibility study
Countries of recruitment	England
Health condition(s) or problem(s) studied	Coronary heart disease
Intervention(s)	Intervention group: initial exercise consultation; personalised exercise prescription delivered via a Polar Ignite watch; monitoring via check-ins and feedback text-message support. Control group: usual care
Key inclusion and exclusion criteria	Inclusion: Coronary heart disease; Completed a community centre-based CR programme; clinically stable (symptoms and medication), access to a smartphone with Bluetooth capacity or a computer/laptop/tablet; able to walk unaided; able to provide informed consent. Exclusion: Absolute contra-indications to exercise as per international clinical guidelines ^31^; Any serious mental health/cognitive issue that will prevent engagement with study procedures or increase the risk of exercise complications; atrial fibrillation or other arrhythmia preventing accurate heart rate monitoring; allergies to the watch materials
Study type	Type: feasibility, interventional, single centre Allocation: randomised Assignment: parallel Masking: outcomes assessor
Date of first enrolment	TBC
Target sample size	30
Recruitment status	Ready to start recruitment
Primary outcome(s)	Feasibility and process-related outcomes: number of patients screened, eligible, recruited, randomised, withdrawn, and retained; adherence to a remotely prescribed and monitored exercise programme; physiological, clinical, and patient-reported outcomes to identify a primary outcome for a definitive trial; acceptability of the interventions and trial procedures via qualitative interviews
Key secondary outcomes	At baseline, three-, six- and 12-months post-randomisation: exercise capacity – incremental shuttle walk test (ISWT); physical activity and sedentary behaviour – Actigraph GT3X + ; cardiovascular disease risk – SMART (Second Manifestations of ARTerial disease) risk score (history, smoking, blood pressure, full lipid profile, estimated glomerular filtration rate (eGFR), and high-sensitivity C-reactive protein (hsCRP); HRQoL – MacNew Quality of Life Questionnaire; depression – Patient Health Questionnaire (PHQ-9); anxiety – Generalised Anxiety Disorder 7-Item Scale (GAD-7); self-efficacy – General Self-Efficacy Scale (GSE); activation – patient Activation Measure (PAM); health utility – EQ-5D-5L; health and social care resource use – Client Service Receipt Inventory (CSRI); adverse and serious adverse events. Monthly: Exercise - Godin Leisure Time Exercise Questionnaire

### Eligibility criteria

Table 1 presents the inclusion/exclusion criteria for people with CHD that are eligible to participate in the planned feasibility RCT.

### Participant identification, recruitment, and informed consent

Potential participants will be identified from existing supervised or home-based CR attendees at UHCW NHS Trust by the clinical team on site (Figure 1). Screening logs will be completed when assessing eligibility, and reason(s) for non-participation will be recorded beginning on the 30^th^ of March 2022 until 30 participants are randomised. Potential participants will be approached by the clinical team either in person, or via phone call, email, or post. Those who are interested will be given information about the study verbally and in a printed participant information leaflet. Once willingness to participate has been confirmed, the participant will be asked to attend a baseline assessment to confirm eligibility and complete written informed consent.

### Randomisation, allocation concealment, and blinding

Following completion of all baseline outcome measures, participants will be randomised to the intervention or control arm on a 1:1 ratio using a computer-validated randomisation system (Sealed Envelope Ltd, www.sealedenvelope.com) independent of the researchers to maintain allocation concealment according to the SPIRIT guidelines.^25^ The MAINTAIN intervention and semi-structured interviews will be completed by a researcher (FD) who will not be blinded to group allocation. All follow-up outcomes will be collected by research staff (AW) not involved in intervention delivery and will be blinded to group allocation. Participants will be asked not to inform the follow-up outcome assessor of their allocation.

### Control intervention: usual care

Participants randomised to usual care will not receive any trial-related treatment or support. Usual care at a CR exit assessment will involve an agreed exercise and physical activity plan from an exercise physiologist to maintain their lifestyle behaviour changes. After completing all the follow-up assessments, however, they will be offered a wearable activity device used in this RCT (Polar Ignite©, Polar Electro Oy, Kempele, Finland) along with the device instruction manual to keep.

### The MAINTAIN intervention

**Figure 2.**
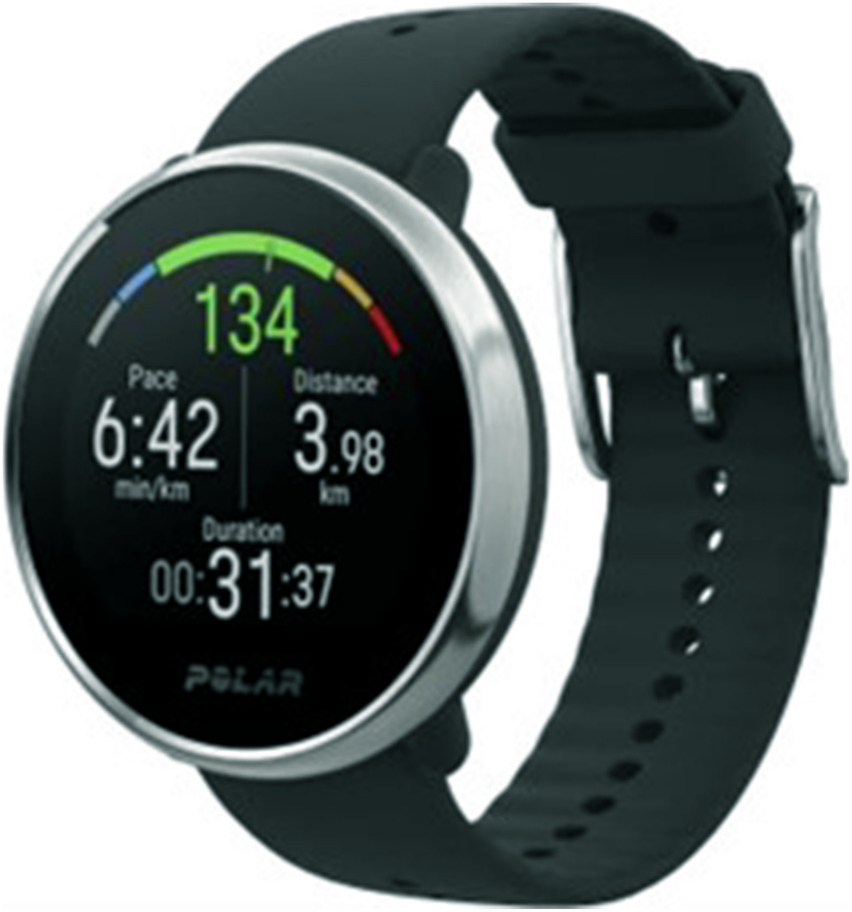
The polar ignite watch used in the intervention arm.^32,33^.

*Pre-intervention.* Participants randomised to the intervention arm will receive a participant pack by post including a Polar Ignite watch (figure 2) and set up instructions to download the ‘Polar Flow- Sync & Analyze’ mobile application or the ‘Polar FlowSync’ data transfer software (https://flow.polar.com/start). Participants will log into an account created by the researcher using the participant's email address and password known only to the participant and researcher. Participants will link their ‘Polar flow’ account to the ‘Flow for Coach’ group to allow the researchers to remotely prescribe and monitor PA and exercise. To extract participant data from the watch, Polar accounts will be linked to the research team using an application programming interface.

*Exercise Consultation.* Each participant in the intervention arm will meet (either in person or via video call) with a clinical exercise physiologist to plan their exercise prescription in accordance with clinical guidelines and personal preferences.^34^ The Polar Ignite^©^ and Polar Flow application functions will be explained and demonstrated to ensure participants are confident using the technology. They will also be signposted to behaviour change resources and diagnosis-specific educational information from the British Heart Foundation (https://www.bhf.org.uk/informationsupport). Following this consultation, the exercise physiologist will schedule the participant's agreed prescription into the online platform.

*Exercise prescription, delivery, and biometric monitoring*. Prescribed exercise sessions will be visible to the participant on the provided fitness watch and on the web or application service. Participants who will perform bodyweight or resistance exercise will be sent guidance using Physiotec (Physiotec HEP, 1996-2022), accessible via an email link, or printed, to ensure exercises are completed safely and effectively without supervision. Participants will be advised to follow the agreed exercise programme and adhere to the watch's haptic vibration feedback to guide heart rate and duration. All data produced during the sessions will be stored on the watch for subsequent synching to the polar flow platform following each exercise session.

*Individualised exercise prescription.* For participants in the intervention arm, exercise prescriptions will be personalised based on outcome assessments, exit assessments of the community CR programme, and exercise data collected during CR. Target heart rates will be based on heart rate prescriptions followed during the participant's phase III CR, calculated by taking into consideration beta-blocker medication and responses on the 6–20 Borg rating of perceived exertion (RPE) scale to monitor intensity. All exercise sessions will include a warm-up and cool-down. Initially, participants will be advised to exercise at least twice per week, progressing to the recommended ≥3 sessions per week as tolerated in accordance with the Association of Chartered Physiotherapists in CR.^35^

*Check-ins.* At the end of the first-month post-allocation/randomisation, participants will attend a check-in with an exercise physiologist either in person or via video or telephone. Data will be reviewed to progress exercise and PA behaviour through goal setting, whilst troubleshooting any issues. The participant's exercise programme will be updated for months two and three. Subsequent check-ins will take place at the beginning of month four, providing updated goals and exercise prescriptions for months four to six, and at the beginning of month seven, updating goals and exercise prescriptions for months seven to 12 of the intervention. At the final check in, instructions for creating and developing a polar flow training plan, either via a web service or via the Polar Flow application, will be provided to encourage participant independence. Plans to overcome individual barriers will be discussed to create an action plan.

*Remote exercise monitoring.* Participants will be advised to synchronise the watch daily and will be asked to wear the watch according to the manufacturer's recommendations whilst exercising. The watch display will guide participants through exercise sessions, whilst simultaneously collecting the exercise data. Following each exercise session, participants will be instructed to synchronise the watch with the Polar Flow application, rate the perceived load of the session based on an RPE from one to 10 (1 classified as ‘very, very easy’, and 10 classified as ‘maximal’), use a five-point Likert scale to rate how they feel, and provide feedback on the exercise session's intensity, duration, and their enjoyment levels. Participant responses will guide text message feedback.

*Personalised text message feedback*. Personalised text message feedback will be gradually tapered between months one, two-three, four-six, and seven-12. Initially messages will be sent after every session, reducing to weekly, biweekly, and then ceasing, respectively. Two-way conversation will be possible between the exercise physiologist and the participant to troubleshoot any issues. Participants completing ≥2 sessions will receive positive feedback text messages. Those completing one session per week will receive positive feedback, but with additional encouragement to complete ≥2 sessions. Participants who have not completed any sessions over a two-week period will receive a text message asking if they need support, and the exercise prescription will be amended accordingly following breaks in training. Participants will also be able to complete and record additional unprescribed ad-hoc exercise sessions.

*Theoretical behavioural underpinning:* The intervention is designed based on the principles of the COM-B model of behaviour change (capability, opportunity, motivation, and behaviour)^36^ with embedded behaviour change techniques. In the context of this study, capability is addressed by increasing the participant's confidence when completing remotely monitored exercise sessions, and by identifying key barriers and how to overcome them during the initial exercise consultation. The provision of British Heart Foundation educational resources will provide knowledge on risk factors. Opportunity relates to removing the barrier of travelling to a centre to exercise, as well as the provision of feedback text messages and prompts from the watch to exercise. Motivation will be addressed using goal setting and feedback text messages.

### Safety

The exercise physiologist delivering the intervention will be an experienced CR practitioner. The assessments will be performed in a cardiopulmonary rehabilitation centre with appropriately trained staff and emergency equipment. Condition-specific monitoring of exercise responses, as per cardiopulmonary rehabilitation guidelines will reduce and manage risk.^31^ The remotely prescribed and monitored exercise and lifestyle PA will replicate exercise performed during the participant's CR programme.

### Outcome measures

*Primary outcome –* feasibility and process related measures:

1. The number of patients screened, eligible, recruited, randomised, withdrawn, and retained.
2. Adherence to the intervention in relation to the number of prescribed exercise sessions completed at the prescribed intensity/duration.
3. Physiological, clinical, patient-reported outcomes to identify a primary outcome for a future definitive RCT.
4. Acceptability of the intervention and trial procedures via qualitative analysis of semi-structured interviews.

**Figure 3.**
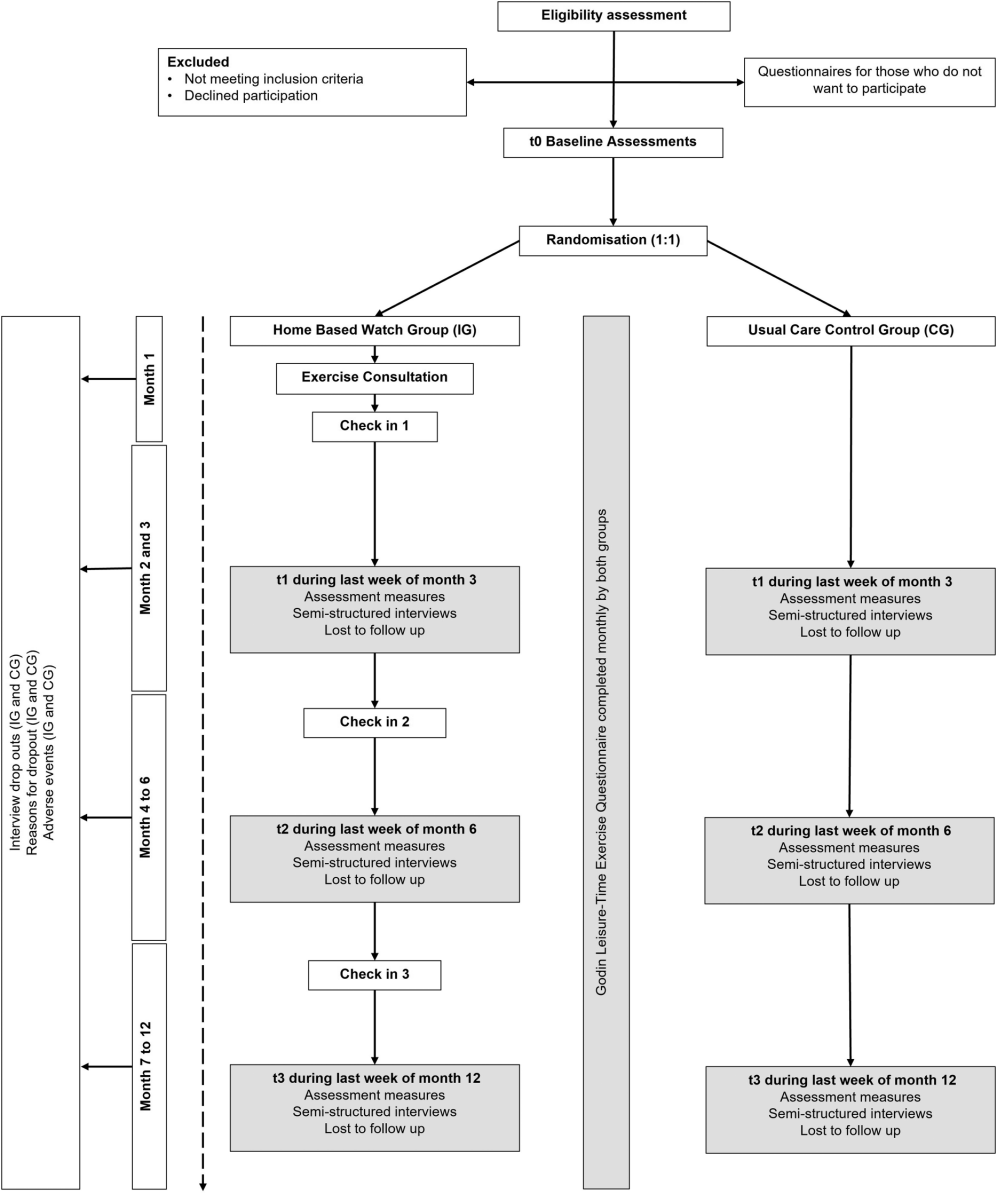
MAINTAIN feasibility randomised controlled trial flow chart.

*Secondary outcomes.* Physiological, clinical, and participant-reported outcomes will be assessed at baseline, and at three-, six-, and 12-months post-randomisation for both the intervention and control arms (Figure 3). Questionnaires will be completed using Qualtrics (Qualtrics, Provo, UT) or a paper version.

### Assessments

Exercise capacity will be assessed with the Incremental Shuttle Walk Test (ISWT).^37^ This involves a 10-m shuttle walk at a steady pace to the timing of an audio track until the participant cannot maintain the required speed. RPE, heart rate and symptoms will be monitored throughout.

PA and sedentary behaviour will be assessed with an Actigraph wGT3X + triaxial accelerometer, worn on a belt around the waist during waking hours for seven days after each follow-up timepoint. This device is reliable in the measurement of PA at various intensities, and sedentary behaviour, during free-living conditions.^38,39^ Exercise time and intensity will be assessed monthly using the Godin Leisure Time Questionnaire (GLTEQ).^40^

Cardiovascular disease risk will be evaluated using the Second Manifestations of ARTerial disease (SMART) risk score.^41^ The score accounts for medical history, smoking status, blood pressure, full lipid profile, estimated filtration rate and high-sensitivity C-reactive protein. Blood samples (5 mL) will be collected using standard venepuncture techniques. Participants will be asked to refrain from participating in exercise for 24 h. Serum will be separated by centrifuging samples at 3000 rpm for 10 min before being aliquoted and frozen at −80 °C. Following analysis samples will be disposed of in accordance with the UK Human Tissue Act.

Disease specific HRQoL will be measured using the MacNew Quality of Life Questionnaire,^42^ which is a reliable and valid questionnaire to assess HRQoL in people with CHD. Depression will be assessed with the Patient Health Questionnaire (PHQ-9)^43^ which is a valid nine item questionnaire,^44^ and anxiety will be measured using the Generalised Anxiety Disorder 7-Item Scale (GAD-7),^45^ which is a self-report questionnaire comprised of seven items, valid for measuring generalised anxiety disorder. Self-efficacy will be assessed using the general self-efficacy scale (GSE),^29^ which is a 10-item psychometric scale designed to assess optimistic self-beliefs to cope with a variety of difficult demands in life. Patient activation, a measure of how able a person can manage their own health, will be assessed with the Patient Activation Measure^®^ (PAM^®^),^46^ a valid and reliable questionnaire useful at an individual level across a range of chronic conditions.

Health utility will be measured using the EQ-5D-5L^47^ questionnaire, which is a validated, generic HRQoL measure consisting of five dimensions to create a health utility score. Health and social care resource use will be assessed using an adapted Client Service Receipt Inventory to examine the feasibility of collecting these data and refine resource use schedules for a definitive RCT. The cost of delivering the intervention will be recorded, including staffing, facility, consumables, etc. To assess the safety of the MAINTAIN trial, adverse events (AEs) and serious adverse events (SAEs) will be monitored in accordance with the principles of Good Clinical Practice.

### Sample size

The MAINTAIN feasibility RCT will assess recruitment rate, adherence, acceptability, and clinical, physiological, and health-related outcomes. The study also aims to identify a primary outcome and estimate a sample size to inform a future definitive RCT based on long-term exercise adherence and health outcomes between the two treatment arms.^48^ However, assuming a medium effect size (0.5) for a repeated measures ANOVA, between two groups over four timepoints with an alpha of 5% and a beta of 90%, a total of 30 participants would be required.^49,50^ Due to the novelty of the proposed outcome measures, a medium effect size will be applied.^51^

From local data (UHCW NHS Trust), 255 patients per year have been identified as accessing community CR of which 212 have a diagnosis of coronary heart disease (CHD). Over a 24-month recruitment period, this provides a potential pool of 510 patients. Local audit data suggests at least 83% of these patients are eligible. Therefore, of these 510 local patients, 423 are potentially eligible, of which we aim to recruit 30 (i.e., approximately 7%, at an approximate rate of five per month).

### Data analysis

Quantitative statistics will be summarised as mean and standard deviation (SD) or median and interquartile range (IQR) for normally and non-normally distributed continuous data, respectively, or frequency and percentage for categorical data at baseline, three-, six-, and 12-month post randomisation by treatment arms. Descriptive statistics will be presented by treatment arms. Data will be assessed for normality using the Shapiro-Wilks test to assess for heterogeneity between groups, baseline values will be compared using independent t-tests or Wilcoxon signed rank tests. Data regarding the number of participants expressing interest, eligible, consented, withdrawn, and lost to follow-up, as well as adherence to remotely prescribed exercise (for intervention arm) and outcomes assessments, will be summarised by treatment arms. Differences in mean outcomes between treatment arms at three-, six-, 12-month post-randomisation, and the difference of the change from baseline to three-, six-, 12-month post-randomisation between arms will be calculated and assessed using a mixed model ANOVA. Assuming the data meet the assumptions of an ANOVA, post hoc tests will be conducted on significant differences. Partial eta squared will be reported as effect sizes. All estimates will be reported with a 95% confidence interval. The alpha level will be set at 0.05. No formal hypothesis testing will be performed. Analysis and reporting of the MAINTAIN trial will be in line with the CONSORT statement.^52^ All primary analyses are planned to be on an intention-to-treat basis with a secondary per protocol analysis. The ADePT (A Process for Decision-making after Pilot and Feasibility Trials)^53^ framework will be used to identify and examine any problems which arise with the methodology. This appraisal of this study will help to find solutions to problems that arise and influence the process of progressing to a definitive RCT. All statistical analysis will be undertaken using IBM SPSS software.

### Embedded qualitative study

Semi-structured interviews will be conducted with 10 participants from both the intervention and control group at three-, six-, and 12-months, and with participants who drop out. The 10 interviewed participants will remain the same throughout all follow-ups. It will not be possible to replace dropouts with additional participants. Interviews will take place via online video call, face-to-face, or phone call, and will be recorded, pseudonymised, and transcribed verbatim. Questionnaires will be offered to those who chose not to participate.

*Qualitative analysis*. Data will be analysed using thematic analysis^54^ in an inductive approach using NVivo^©^ (QSR International Pty Ltd, Chadstone, VIC, Australia) software to allow rich and detailed information regarding the acceptability of the trial, Polar Ignite watch and intervention. Data will be analysed in the following steps:

1. Data familiarisation: reading and listening to complete interview transcripts, audio recordings and field notes.
2. Generate initial codes: transcripts will be coded, and key concepts identified.
3. Searching for themes: codes will generate themes and subthemes as an output from the data to develop an initial thematic map.
4. Reviewing themes: codes included in generated themes will be checked to ensure they create a pattern, and the validity of the themes will be reviewed to ensure they represent the whole data set.
5. Defining and naming themes: each theme will be defined succinctly, and possible subthemes will be reviewed before naming the theme.
6. Producing the report: data will be analysed and written up.

Both qualitative and quantitative data will be synthesised to integrate the overall results. Comparing both data types of data will reveal any differences or agreements, whilst providing an overall representation of the intervention's feasibility.

### Data collection and management

Personal data will be handled and stored in accordance with General Data Protection Regulation and Coventry University's confidentiality and data protection policies. All data will be anonymised, and each participant following randomisation will be designated a unique trial ID. Data will be confidentially stored at UHCW, NHS Trust in a secure location and/or on password-protected computer drive. During the trial, monitoring will be completed frequently by research team members including the CI (FD). Following study completion, data will be archived for up to 5 years before all essential documentation is transferred to a third-party archiving service for a minimum of 3 years after the results have been publicly released or published.

### Adverse event (AE) management

An AE will be defined as any untoward medical occurrence involving a participant, which does not necessarily have a causal relationship with the intervention or trial. Expected AEs related to the exercise outcome assessments or intervention including chest pain, muscle and joint stiffness or soreness, and tiredness/fatigue will be recorded, but not reported. Unexpected AEs will be assessed for relatedness and recorded. Expected SAEs will be recorded, but not reported; these include elective or pre-planned treatment for a pre-existing condition, and general care, both of which should not be associated with any deterioration in condition. Where required, AEs and SAEs will be reported to Coventry University within 24 h of the CI becoming aware. The Research Ethics Committee will be notified of an SAE within 15 days of the CI becoming aware if it is unexpected and possibly, probably, or definitely related to the trial.

### Dissemination

Study results will be published in a peer-reviewed journal, presented at relevant conferences, and provided to participants. Data, results, and sponsor information will not be passed on to third parties without prior consent from the sponsor and participants.

## Discussion

After completion of community CR, long-term exercise, PA adherence and health-outcomes in people with CHD can be poor.^13,14^ Previous research using wearable activity devices in the maintenance phase of CR has reported promising results,^26,27^ however, outcomes have rarely been examined beyond three months. Wearable activity devices supported by exercise prescription may improve everyday PA (number of steps), exercise intensity, cardiorespiratory fitness, and functional capacity in those who adhere, but the effect on sedentary time has not been established.^23,55^ Additionally, evidence is inconclusive as to whether such interventions can improve long-term psychological outcomes. Further primary evidence is still required for peak aerobic capacity, maintaining PA, exercise intensity, and psychological effects in people with CHD.^55^ Qualitative data regarding perceptions and acceptability of remote activity monitoring devices have often not been simultaneously assessed with quantitative data, specifically in the case of the Polar Ignite device. Therefore, further research is warranted in this field to understand the clinical and cost-effectiveness of remotely prescribed and monitored exercise maintenance programmes following CR and to evaluate adherence to PA/exercise in the long-term.

Developing new options for patients to continue long-term exercise and PA, may also infiltrate the National Health Service's Long-Term Plan of increasing CR provision to accommodate 85% of eligible patients by 2028.^56^ Remotely prescribed and monitored mHealth programmes could overcome key barriers associated with centre-based CR and, therefore, be adopted, not only for long-term maintenance programmes, but as an alternative to early CR supervised centre-based programmes. Remote maintenance CR programs may also help patients with CHD to transition to a new routine in the home environment or provide an alternative option for patients at high risk of COVID-19 severe illness.^57^ Therefore, if the intervention proves to be useful in supporting people after CR, further research could investigate its implementation in wider practice such as exercise referral schemes or for people with other long-term conditions.

The MAINTAIN trial aims to examine the feasibility of a long-term exercise and PA maintenance programme, which is remotely prescribed and monitored using a wearable activity device with real-time biometrics and haptic feedback, alongside personalised text messaging in people with CHD. MAINTAIN will address PA behaviours, exercise adherence, and a variety of health-outcomes over a 12-month period. The study will also aim to identify a primary outcome for a future definitive trial and provide additional evidence to fill key gaps in the literature relating to long-term health outcomes.^55^ This feasibility study aims to tackle an important and complex problem by taking a multi-faceted approach to improving long term adherence to exercise after completion of CR. Both qualitative and quantitative data will be collected to examine the acceptability of the intervention, and interviews will help to inform patient and public involvement and engagement for a future definitive RCT. To minimise the effects of both bias and attrition, as reported in a previous systematic review,^55^ the MAINTAIN trial will blind outcomes assessors and apply an intention to treat analysis. Adverse events will be reported according to trial arm.

## References

[1] British Heart Foundation. Heart statistics - Heart and Circulatory Diseases in the UK | BHF, https://www.bhf.org.uk/what-we-do/our-research/heart-statistics (accessed March 28, 2022).

[2] Liu JLY Maniadakis N Gray A, et al. The economic burden of coronary heart disease in the UK. Heart 2002; 88(6): 597–603. 12433888 10.1136/heart.88.6.597 PMC1767465

[3] Mehra VM Gaalema DE Pakosh M, et al. Systematic review of cardiac rehabilitation guidelines: quality and scope. Eur J Prev Cardiol 2020; 27(9): 912–928. 31581808 10.1177/2047487319878958 PMC7262778

[4] NICE. Acute coronary syndromes NICE guideline, www.nice.org.uk/guidance/ng185 (2020, accessed March 28, 2022).

[5] McGregor G Powell R Kimani P, et al. Does contemporary exercise-based cardiac rehabilitation improve quality of life for people with coronary artery disease? A systematic review and meta-analysis. BMJ Open 2020; 10(6): e036089. 10.1136/bmjopen-2019-036089 PMC7282413 32513887

[6] Anderson L Sharp GA Norton RJ, et al. Home-based versus centre-based cardiac rehabilitation. Cochrane Database Syst Rev 2017; 6: CD007130. 28665511 10.1002/14651858.CD007130.pub4 PMC6481471

[7] Thomas RJ Beatty AL Beckie TM, et al. Home-based cardiac rehabilitation: a scientific statement from the American association of cardiovascular and pulmonary rehabilitation, the American heart association, and the American college of cardiology. Circulation 2019; 140(1): E69–E89. 31082266 10.1161/CIR.0000000000000663

[8] Stefanakis M Batalik L Antoniou V, et al. Safety of home-based cardiac rehabilitation: a systematic review. Heart Lung 2022; 55: 117–126. 35533492 10.1016/j.hrtlng.2022.04.016

[9] Snoek JA Prescott EI van der Velde AE, et al. Effectiveness of home-based mobile guided cardiac rehabilitation as alternative strategy for nonparticipation in clinic-based cardiac rehabilitation among elderly patients in Europe: a randomized clinical trial. JAMA Cardiol 2021; 6(4): 463–468. 33112363 10.1001/jamacardio.2020.5218 PMC7593879

[10] Kabboul NN Tomlinson G Francis TA, et al. Comparative effectiveness of the core components of cardiac rehabilitation on mortality and morbidity: a systematic review and network meta-analysis. J Clin Med 2018; 7(12): 514. 30518047 10.3390/jcm7120514 PMC6306907

[11] Abreu A Frederix I Dendale P, et al. Standardization and quality improvement of secondary prevention through cardiovascular rehabilitation programmes in Europe: the avenue towards EAPC accreditation programme: a position statement of the secondary prevention and rehabilitation section of the European association of preventive cardiology (EAPC). Eur J Prev Cardiol 2021; 28(5): 496–509. 10.1177/2047487320924912 33611459

[12] Giannuzzi P Mezzani A Saner H, et al. Physical activity for primary and secondary prevention. Position paper of the working group on cardiac rehabilitation and exercise physiology of the European society of cardiology. Eur J Prev Cardiol 2003; 10(5): 319–327. 10.1097/01.hjr.0000086303.28200.50 14663293

[13] Bock BC Carmona-barros RE Esler JL, et al. Program participation and physical activity maintenance. Behav Modif 2003; 27(1): 37–53. 12587259 10.1177/0145445502238692

[14] Dolansky MA Stepanczuk B Charvat JM, et al. Women’s and men’s exercise adherence after a cardiac event. Res Gerontol Nurs 2010; 3(1): 30–38. 20128541 10.3928/19404921-20090706-03 PMC2897096

[15] Horwood H Williams MJA Mandic S. Examining motivations and barriers for attending maintenance community-based cardiac rehabilitation using the health-belief model. Heart Lung Circ 2015; 24(10): 980–987. 25939724 10.1016/j.hlc.2015.03.023

[16] Stockwell S Trott M Tully M, et al. Changes in physical activity and sedentary behaviours from before to during the COVID-19 pandemic lockdown: a systematic review. BMJ Open Sport Exerc Med 2021; 7(1): e000960. 10.1136/bmjsem-2020-000960 PMC7852071 34192010

[17] Fersia O Bryant S Nicholson R, et al. The impact of the COVID-19 pandemic on cardiology services. Open Heart 2020; 7(2): e001359. 32855212 10.1136/openhrt-2020-001359 PMC7454176

[18] Denton F Power S Waddell A, et al. Is it really home-based? A commentary on the necessity for accurate definitions across exercise and physical activity programmes. Int J Environ Res Public Health 2021; 18(17): 9244. 34501833 10.3390/ijerph18179244 PMC8431042

[19] O’Doherty AF Humphreys H Dawkes S, et al. How has technology been used to deliver cardiac rehabilitation during the COVID-19 pandemic? An international cross-sectional survey of healthcare professionals conducted by the BACPR. BMJ Open 2021; 11(4): e046051. 10.1136/bmjopen-2020-046051 PMC8061561 33879492

[20] Falter M Scherrenberg M Dendale P. Digital health in cardiac rehabilitation and secondary prevention: a search for the ideal tool. Sensors (Switzerland) 2021; 21(12): 1–11. 10.3390/s21010012 PMC7792579 33374985

[21] Antoniou V Davos CH Kapreli E, et al. Effectiveness of home-based cardiac rehabilitation, using wearable sensors, as a multicomponent, cutting-edge intervention: a systematic review and meta-analysis. J. Clin Med 2022; 11(13): 3772. 10.3390/JCM11133772/S1 35807055 PMC9267864

[22] Franssen WMA Franssen GHLM Spaas J, et al. Can consumer wearable activity tracker-based interventions improve physical activity and cardiometabolic health in patients with chronic diseases? A systematic review and meta-analysis of randomised controlled trials. Int J Behav Nutr Phys Act 2020 May 11; 17: 57. 32393357 10.1186/s12966-020-00955-2 PMC7216601

[23] Kirk MA Amiri M Pirbaglou M, et al. Wearable technology and physical activity behavior change in adults with chronic cardiometabolic disease: a systematic review and meta-analysis. Am J Health Promot 2019; 33(5): 778–791. 30586996 10.1177/0890117118816278

[24] Laranjo L Ding D Heleno B, et al. Do smartphone applications and activity trackers increase physical activity in adults? Systematic review, meta-analysis and metaregression. Br J Sports Med 2020; 0: 1–13. 10.1136/bjsports-2020-102892 33355160

[25] Lunde P Bye A Bergland A, et al. Long-term follow-up with a smartphone application improves exercise capacity post cardiac rehabilitation: a randomized controlled trial. Eur J Prev Cardiol 2020; 27(16): 1782–1792. 32106713 10.1177/2047487320905717 PMC7564298

[26] Park LG Elnaggar A Lee SJ, et al. Mobile health intervention promoting physical activity in adults post cardiac rehabilitation: pilot randomized controlled trial. JMIR Form Res 2021; 5(4): e20468. 33861204 10.2196/20468 PMC8087971

[27] Duscha BD Piner LW Patel MP, et al. Effects of a 12-week mHealth program on peak VO 2 and physical activity patterns after completing cardiac rehabilitation: a randomized controlled trial. Am Heart J 2018; 199: 105–114. 29754647 10.1016/j.ahj.2018.02.001

[28] Albergoni A Hettinga FJ La Torre A, et al. The role of technology in adherence to physical activity programs in patients with chronic diseases experiencing fatigue: a systematic review. Sports Med Open 2019; 5: 41. 31512075 10.1186/s40798-019-0214-z PMC6739434

[29] Schwarzer R Jerusalem M Weinman J, et al. Measures in health psychology: a user’s portfolio. Causal Control Beliefs 1995; 1.

[30] Chan A-W Tetzlaff JM Altman DG, et al. SPIRIT 2013 Statement: defining standard protocol items for clinical trials. Ann Intern Med 2013; 158(3): 200. 23295957 10.7326/0003-4819-158-3-201302050-00583 PMC5114123

[31] American College of Sports Medicine. ACSM’s Guidelines for exercise testing and prescription. 10th ed. Riverwoods, IL: Lippincott Williams & Wilkins, 2017, 10th ed. Philadelphia: Wolters Kluwer, 2018.

[32] Polar. Polar Ignite Fitness Watch, https://www.polar.com/uk-en/ignite/?wgu = 293045_1347475_16709457216353_b856fdae66&wgexpiry = 1702481721&source = webgains&siteid = 1347475 (accessed 13 December 2022).

[33] Polar Store. Polar Ignite - Fitness Watch with Advanced Wrist-Based Optical Heart rate Monitor, Training Guide, GPS, Waterproof, https://www.amazon.co.uk/Fitness-Advanced-Wrist-Based-Training-Waterproof/dp/B07T3GTHFY?source = ps-sl-shoppingads-lpcontext&ref_ = fplfs&smid = A3P5ROKL5A1OLE&th = 1&psc = 1 (accessed 13 December 2022).

[34] Jones H George KP Scott A, et al. Charter to establish clinical exercise physiology as a recognised allied health profession in the UK: a call to action. BMJ Open SEM 2021; 7(3): e001158. 10.1136/bmjsem-2021-001158 PMC8458347 34631147

[35] Association of Chartered Physiotherapists in Cardiac Rehabilitation. ACPICR Standards Standards for Physical Activity and Exercise in the Cardiovascular Population. 3rd ed., https://www.acpicr.com/data/Page_Downloads/ACPICRStandards.pdf (2015, accessed 15 December 2022).

[36] Michie S van Stralen MM West R. The behaviour change wheel: a new method for characterising and designing behaviour change interventions. Implement Sci 2011; 6(1): 42. 21513547 10.1186/1748-5908-6-42 PMC3096582

[37] Singh SJ Morgan MDL Scott S, et al. Development of a shuttle walking test of disability in patients with chronic airways obstruction. Thorax 1992; 47: 1019–1024. 1494764 10.1136/thx.47.12.1019 PMC1021093

[38] Aadland E Ylvisåker E. Reliability of the actigraph GT3X + accelerometer in adults under free-living conditions. PLoS One 2016; 10(8): e0134606. 10.1371/journal.pone.0134606 PMC4537282 26274586

[39] Kim Y Barry VW Kang M. Validation of the ActiGraph GT3X and activPAL accelerometers for the assessment of sedentary behavior. Meas Phys Educ Exerc Sci 2015; 19(3): 125–137.

[40] Godin G Shephard RJ. A simple method to assess exercise behavior in the community. Can J Appl Sci 1985; 10(3): 141–146. 4053261

[41] Dorresteijn JAN Visseren FLJ Wassink AMJ, et al. Development and validation of a prediction rule for recurrent vascular events based on a cohort study of patients with arterial disease: the SMART risk score. Heart 2013; 99(12): 866–872. 23574971 10.1136/heartjnl-2013-303640

[42] Oldrige N Guyatt G Jones N, et al. Effects on quality of life with comprehensive rehabilitation after acute myocardial infarction. Am J Cardiol 1991; 67(13): 1084–1089. 2024598 10.1016/0002-9149(91)90870-q

[43] Spitzer RL Kroenke K Williams JBW. Validation and utility of a self-report version of PRIME-MD: the PHQ primary care study. JAMA 1999; 282(218): 1737–1744. 10568646 10.1001/jama.282.18.1737

[44] Kroenke K Spitzer RL Williams JBW. The PHQ-9: validity of a brief depression severity measure. J Gen Intern Med 2001; 16(9): 606–613. 11556941 10.1046/j.1525-1497.2001.016009606.x PMC1495268

[45] Spitzer RL Kroenke K Williams JBW, et al. A brief measure for assessing generalized anxiety disorder: the GAD-7. Arch Intern Med 2006; 166(9): 1092–1097. 16717171 10.1001/archinte.166.10.1092

[46] Hibbard JH Stockard J Mahoney ER, et al. Development of the patient activation measure (PAM): conceptualizing and measuring activation in patients and consumers. Health Serv Res 2004; 39: 1005–1026. 15230939 10.1111/j.1475-6773.2004.00269.x PMC1361049

[47] Herdman M Gudex C Lloyd A, et al. Development and preliminary testing of the new five-level version of EQ-5D (EQ-5D-5L). Qual Life Res 2011; 20(10): 1727–1736. 21479777 10.1007/s11136-011-9903-x PMC3220807

[48] Eldridge SM Chan CL Campbell MJ, et al. CONSORT 2010 Statement: extension to randomised pilot and feasibility trials. Pilot Feasibility Stud 2016; 2(1): 1–32. 27965879 10.1186/s40814-016-0105-8 PMC5154046

[49] Whitehead AL Julious SA Cooper CL, et al. Estimating the sample size for a pilot randomised trial to minimise the overall trial sample size for the external pilot and main trial for a continuous outcome variable. Stat Methods Med Res 2016; 25(3): 1057–1073. 26092476 10.1177/0962280215588241 PMC4876429

[50] Faul F Erdfelder E Lang AG, et al. G*power 3: a flexible statistical power analysis program for the social, behavioral, and biomedical sciences. Behav Res Methods 2007; 39(2): 175–191. 17695343 10.3758/bf03193146

[51] Browne RH. On the use of a pilot sample for sample size determination. Stat Med 1995; 14(17): 1933–1940. 8532986 10.1002/sim.4780141709

[52] Schulz KF Altman DG Moher D. CONSORT 2010 Statement: updated guidelines for reporting parallel group randomised trials. Br Med J 2010; 340: 698–702. 10.4103/0976-500X.72352 PMC3043330 21350618

[53] Bugge C Williams B Hagen S, et al. A process for decision-making after pilot and feasibility trials (ADePT): development following a feasibility study of a complex intervention for pelvic organ prolapse. Trials 2013; 14(1): 353. 24160371 10.1186/1745-6215-14-353 PMC3819659

[54] Braun V Clarke V. Using thematic analysis in psychology. Qual Res Psychol 2006; 3(2): 77–101.

[55] Hannan AL Harders MP Hing W, et al. Impact of wearable physical activity monitoring devices with exercise prescription or advice in the maintenance phase of cardiac rehabilitation: systematic review and meta-analysis. BMC Sports Sci Med Rehabil 2019; 11(1): 1–21. 31384474 10.1186/s13102-019-0126-8 PMC6668165

[56] The NHS Long Term Plan. www.longtermplan.nhs.uk (2019, accessed March 28, 2022).

[57] Pepera G Tribali MS Batalik L, et al. Epidemiology, risk factors and prognosis of cardiovascular disease in the coronavirus disease 2019 (COVID-19) pandemic era: a systematic review. Rev Cardiovasc Med 2022; 23(1): 28. 35092220 10.31083/j.rcm2301028

